# Molecular Decoy to the Y-Box Binding Protein-1 Suppresses the Growth of Breast and Prostate Cancer Cells whilst Sparing Normal Cell Viability

**DOI:** 10.1371/journal.pone.0012661

**Published:** 2010-09-10

**Authors:** Jennifer H. Law, Yvonne Li, Karen To, Michelle Wang, Arezoo Astanehe, Karen Lambie, Jaspreet Dhillon, Steven J. M. Jones, Martin E. Gleave, Connie J. Eaves, Sandra E. Dunn

**Affiliations:** 1 Laboratory for Oncogenomic Research, Child and Family Research Institute, University of British Columbia, Vancouver, British Columbia, Canada; 2 Genome Sciences Centre, BC Cancer Agency, Vancouver, British Columbia, Canada; 3 The Prostate Centre, Vancouver General Hospital, University of British Columbia, Vancouver, British Columbia, Canada; 4 Terry Fox Laboratories, BC Cancer Agency, University of British Columbia, Vancouver, British Columbia, Canada; University Medical Center Utrecht, Netherlands

## Abstract

The Y-box binding protein-1 (YB-1) is an oncogenic transcription/translation factor that is activated by phosphorylation at S102 whereby it induces the expression of growth promoting genes such as EGFR and HER-2. We recently illustrated by an *in vitro* kinase assay that a novel peptide to YB-1 was highly phosphorylated by the serine/threonine p90 S6 kinases RSK-1 and RSK-2, and to a lesser degree PKCα and AKT. Herein, we sought to develop this decoy cell permeable peptide (CPP) as a cancer therapeutic. This 9-mer was designed as an interference peptide that would prevent endogenous YB-1^S102^ phosphorylation based on molecular docking. In cancer cells, the CPP blocked P-YB-1^S102^ and down-regulated both HER-2 and EGFR transcript level and protein expression. Further, the CPP prevented YB-1 from binding to the EGFR promoter in a gel shift assay. Notably, the growth of breast (SUM149, MDA-MB-453, AU565) and prostate (PC3, LNCap) cancer cells was inhibited by ∼90% with the CPP. Further, treatment with this peptide enhanced sensitivity and overcame resistance to trastuzumab in cells expressing amplified HER-2. By contrast, the CPP had no inhibitory effect on the growth of normal immortalized breast epithelial (184htert) cells, primary breast epithelial cells, nor did it inhibit differentiation of hematopoietic progenitors. These data collectively suggest that the CPP is a novel approach to suppressing the growth of cancer cells while sparing normal cells and thereby establishes a proof-of-concept that blocking YB-1 activation is a new course of cancer therapeutics.

## Introduction

Cancer is a leading cause of death worldwide, with the World Health Organization estimating 7.9 million deaths annually in 2007. The major cause of mortality in these patients can be attributed to cancers that are resistant to current therapies either intrinsically or through acquired mechanisms. In the case of breast cancer, ∼207,000 new cases will be diagnosed annually in the US and Canada and ∼46,000 women will die from this disease [1], [2]. There are 5 subtypes of breast cancer and the mortality rates differ depending on the subtype, with the triple-negative and HER-2 positive subtypes having the poorest prognoses [3]. The triple-negative subtype, for example, does not respond to conventional anti-hormone or molecular targeting therapies (i.e. trastuzumab) and therefore has limited treatment options. Similarly, in the HER-2 positive subtype, only 30–50% of patients respond to trastuzumab and even then, these patients often develop resistance over time [4], [5]. For prostate cancer, an estimated 211,000 men will be diagnosed with prostate cancer this year in North America and 33,000 men will die from the disease [1], [2]. Like breast cancer, traditional hormone therapies are not effective in androgen-independent prostate cancer and resistance to current therapies is also common [6]–[8]. As a result, these subtypes of breast and prostate cancer represent a significant unmet medical need.

Y-box binding protein-1 (YB-1) is a transcription and translation factor that alters the expression of at least ten genes strongly linked to drug resistance and tumour cell growth such as the epidermal growth factor receptor (EGFR) and the human epidermal growth factor receptor-2 (HER-2). YB-1 has multiple effects on cancer cells [9], with over-expression leading to increased proliferation and siRNA silencing inhibiting growth and inducing apoptosis. YB-1 has been shown to be highly expressed in many cancers, including breast [10], [11], prostate [12], bone [13], lung [14], [15], and colon [16]. YB-1 is also over-expressed in a large proportion of brain tumours affecting adults and children [17], [18] whereby it underpins drug resistance to classically administered drugs such as temozolamide [18]. We have demonstrated that this protein was a strong predictive factor for poor overall survival in breast cancer patients [11] and recently, another group has shown that YB-1 is a predictive marker of prognosis in non-small cell lung cancer [19]. Further, YB-1 is preferentially expressed in cancers over normal adult tissues. Thus, we have long been interested in targeting YB-1 for cancer therapeutics.

Previous work in our lab showed that the serine/threonine kinase AKT phosphorylates, and thereby activates, YB-1 at S102 and that inhibiting this site disrupts nuclear trafficking [20], DNA binding [10] and tumour cell growth [20]. More recently, we determined that p90 ribosomal S6 kinase (RSK) is the predominate kinase that phosphorylates YB-1 at S102, and that PKCα and AKT do so to a lesser degree [21]. Although relatively little is known about the specific role that RSK plays in cancer, it has been reported to be over-expressed in breast and prostate cancers, and has an important role in the MAPK pathway, cell survival, and proliferation [21]–[24]. We therefore designed a YB-1 cell permeable peptide (CPP) to compete for phosphorylation at S102 by RSK. Since this peptide mimics the crucial activation site of YB-1, it should also block endogenous phosphorylation of YB-1 by AKT and PKCα. Thus, in theory, increasing concentrations of the interference peptide will decrease the phosphorylation of wild-type YB-1 and prevent it from binding to growth promoting/resistance genes. This approach possesses several advantages: 1) Signal transduction inhibitors as molecular targeting therapies for cancer have been clinically validated with compounds such as trastuzumab (Herceptin®), imatinib mesylate (Gleevec®) and gefitinib (Iressa®), 2) CPPs have the potential to provide therapeutic options for triple-negative and trastuzumab-resistant breast cancer as well as prostate cancer, and, 3) Since the YB-1 pathway has been implicated in many different cancer types, the technology has broad utility.

We report here that the CPP was readily taken up by breast cancer cells, inhibited phosphorylation of YB-1, and reduced growth in a dose-dependant manner in models of incurable breast cancer (triple-negative and HER-2+). These results correlated with demonstrated suppression of EGFR and HER-2 protein and transcript levels that are two YB-1 target genes. We found similar results in androgen-independent (PC3) and dependent (LNCap) prostate cancer cell lines. Further, the peptide had little effect on the growth of normal breast epithelial cells or hematopoietic progenitors. We therefore establish a proof-of-principle that blocking P-YB-1^S102^ is a potential axis for cancer therapeutics.

## Materials and Methods

### Cell permeable peptide development

The cell permeable peptide (CPP) was developed from the YB-1 peptide sequence containing the S102 site, PRKYLRSVG, and was linked to antennapedia, a 16mer amino acid sequence (RQIKIWFQNRRMKWKK) that facilitates peptide delivery into cells [25]–[27]. The control peptide, RALKYGVRP, was a scrambled sequence of the CPP and was also linked to antennapedia (referred to as scrambled peptide henceforth). Initially, these peptides were biotin labeled and visualized with Oregon Green 514 (Gibco/Invitrogen, Burlington, ON) to show that they entered the cell. Once determined, further experiments were performed without the biotin label. The peptides were custom synthesized by thinkpeptide (ProImmune, Oxford, UK) and dissolved in 3% acetic acid to a stock concentration of 25 mM.

### Modeling phosphorylation of YB-1 by RSK

A model of the RSK-CPP-binding complex was built on a known binding complex of AKT-GSK3β (PDB id 1o6k) [28], using the Molsoft ICM 3.5-1m homology modeling and docking packages [29]. We chose not to use existing RSK crystal structures, as they possessed a 12-residue gap of a loop structure near the peptide-binding site. First a homology model of RSK was built using AKT as a reference (46% sequence identity over the kinase domain and 67% within residues 3.5 Å to the peptide-binding site). The CPP was then docked into the peptide-binding site of RSK, defined as a box 3.5 Å around the known GSK3β peptide-binding site. With the supposition that the CPP would be phosphorylated at the serine site (S102 in the complete YB-1 protein [20]), we visually scanned through the list of docked conformations and selected those with the serine residue in a position allowing for phosphorylation. These conformations served as starting positions for further docking, each time selecting a peptide with similar or better ICM docking score and a superior binding conformation by backbone alignment to the known GSK3β peptide conformation. The process concluded when the binding poses and energies of the selected peptide conformation stabilized over 5 iterations.

### Cell culture

All cell lines were obtained from the American Tissue Culture Collection (Manassus, VA) unless otherwise stated. Human mammary epithelial cells (184htert, gift from Dr. J. Carl Barrett, National Institutes of Health, Bethesda, MD) and the breast cancer cell lines SUM149 (Asterand, Ann Arbor, MI), AU565, BT474, BT474-m1 (a metastatic variant of BT474, gift from Dr. Mien-Chi Hung, MD Anderson Cancer Center, Houston, TX), and MDA-MB-453 were grown as previously described [30]–[33]. The HR5 and HR6's are trastuzumab resistant cell lines [34] obtained from Dr. Carlos Arteaga (Vanderbilt-Ingram Cancer Center, Nashville, TN). Prostate cancer PC3 and LNCap cells were grown in RPMI-1640 plus 10% FBS (Gibco/Invitrogen). All cells were grown at 37°C with 5% CO_2_.

### Oregon Green staining

SUM149 cells were plated at 3000 cells/well in a 96 well plate and treated with DMSO, 1.25 µM biotin-labeled scrambled peptide (Biotin-RQIKIWFQNRRMKWKKRALKYGVRP -OH) or 1.25 µM CPP (Biotin-RRQIKIWFQNRRMKWKKPRKYLRSVG-OH) for 1 h at 37°C. The media was removed and cells were fixed for 20 min in 2% paraformaldehyde/PBS, washed with PBS and permeabilized with 0.1% Triton-X 100 for 10 min. Cells were then incubated with 1/5000 dilution of Oregon Green 514 (Invitrogen) for 1 h, washed with PBS and stained with 0.5 µg/mL Hoechst (Sigma Chemical, Oakville, ON) for 3 min. The ability of the peptides to enter the cell was analyzed on an Arrayscan VTI Reader (Cellomics, Pittsburgh, PA).

### Immunoblotting

Cell lines used herein were evaluated for YB-1, the activating kinases RSK and AKT, and the downstream targets EGFR and HER-2. To demonstrate that phophorylation of YB-1 was decreased with CPP treatment, cells were treated with either 50 µM scrambled CPP or 12.5–50 µM of the CPP for 72 h. Proteins were extracted using ELB plus protease and phosphatase inhibitors and western blotting was performed as previously described [10]. The following antibodies were used to detect proteins by immunoblotting (unless otherwise indicated, all antibodies were from Cell Signaling Technology, Danvers, MA): P-YB-1^S102^ (1/500), YB-1 (1/2000, Abcam, Cambridge, MA), EGFR (1/5000, Stressgen, San Diego, CA), P-p90RSK^S380^ (1/500), RSK1/RSK2/RSK3 (1/1000), HER-2 (1/1000, Abcam), AKT (1/1000), creb (1/1000), pan-actin (1/1000), vinculin (1/1000). Blots were quantified using ImageJ software.

### Real-time quantitative reverse transcription PCR

RNA was isolated from log growing AU565 and SUM149 cells treated with 50 µM scrambled CPP or 50 µM CPP for 24 h using the RNeasy Mini kit from Qiagen (Mississauga, ON). The RNA was reverse transcribed and amplified using HER-2 (for AU565 cells) or EGFR (for SUM149 and AU565 cells) specific primers and probes (Applied Biosystems, Foster City, CA). 18S mRNA was quantified as a housekeeping gene (Applied Biosystems).

### Electrophoretic mobility shift assay (EMSA)

The gel shift assay was performed using EGFR2a oligonucleotides as previously described [31]. Briefly, SUM149 cells were harvested and cytoplasmic/nuclear fractions were obtained using the NE-PER kit followed by EMSA using the Lightshift Chemiluminescent EMSA kit (both kits from Pierce Biotechnology, Rockford, IL). Nuclear extracts were confirmed to contain RSK and YB-1 by immunoblotting. Nuclear protein (5 µg) was pre-incubated for 20 min with unlabeled oligonucleotide plus acetic acid control, scrambled peptide (25 µM), or CPP (25 µM or 50 µM) prior to the addition of biotin-labeled oligonucleotide. Chicken anti-YB-1 (1 µg) was used as positive control. Reactions were run on a non-denaturing polyacrylamide gel, transferred to a nylon membrane, cross-linked at 120 mJ/cm^2^ using a Stratalinker (Stratagene, La Jolla, CA) and visualized with chemiluminescence (Pierce Biotechnology).

### Growth assay

For monolayer assays, cells were plated at 5000 cells per well in a 96 well plate. The following day cells were treated with diluent, 0–50 µM of scrambled peptide, or 0–50 µM CPP. The BT474, BT474-m1, HR5 and HR6 cells were also tested in combination with trastuzumab (BC Cancer Agency pharmacy, Vancouver, BC) at 20 µg/mL. After 72 h treatment, cell growth was analyzed by MTS for all cell lines except the AU565 cells. In the case of these cells, MTS did not provide an accurate measurement of growth and therefore the AU565 cells were stained with Hoechst and counted on the Arrayscan VTI Reader (Cellomics) as previously described [32]. Growth was shown as a percentage of diluent control and changes in growth were considered significant at P<0.01.

### Normal mammary epithelial cells assay

EpCAM^+^ primary normal human breast epithelial cells from two patients were obtained, prepared, and isolated as previously described [35], [36]. In all subsequent experiments, the primary epithelial cells were grown in Epicult-B media (StemCell Technologies, Vancouver, BC) supplemented with 5% fetal calf serum. For the colony-forming cell (CFC) assay, EpCAM^+^ cells from each patient were first seeded at 7000 cells/well into a 96 well plate and treated the following day with either diluent, scrambled peptide (6 or 25 µM), or CPP (6 or 25 µM) in triplicate wells for 48 hours. Following this peptide incubation, cells were harvested with trypsin, a single cell suspension was generated, and cells were seeded into the CFC assay as previously described [36], [37]. Colonies were counted 7 days post-seeding. EpCAM^+^ cells from each patient were also plated into a 96 well plate (1000 cells/well), treated the following day for 72 h with the same peptide treatments as above, and analyzed for growth using an MTS assay.

### Hematopoietic progenitor assay

Thawed normal bone marrow cells were enriched for CD34+ cells using an immunomagnetic positive selection method (EasySep, StemCell Technologies) according to the manufacturer's instructions (CD34+ purity  = 94%). To measure the toxicity of the YB-1 inhibitor peptide on the colony-forming cell hematopoietic progenitors, CFC assays were set up with varying concentrations of CPP (0–50 µM). The negative scrambled peptide and solvent control were also included in the experiment. Aliquots of the cells were plated in Methocult H4435 (StemCell Technologies) that contains erythropoietin, steel factor, interleukin-3, interleukin-6, granulocyte colony stimulating factor and granulocyte macrophage colony stimulating factor. Erythroid colonies (BFU-Es), granulopoietic colonies (CFU-GMs) and multi-lineage colonies (CFU-GEMM) were scored by direct visualization using an inverted microscope (32×) 14 days later using standard criteria [38]. These values were then added together to obtain total CFC counts per cells plated and expressed as % of control (the 0 µM YB-1 assay).

## Results and Discussion

**Figure 1:**
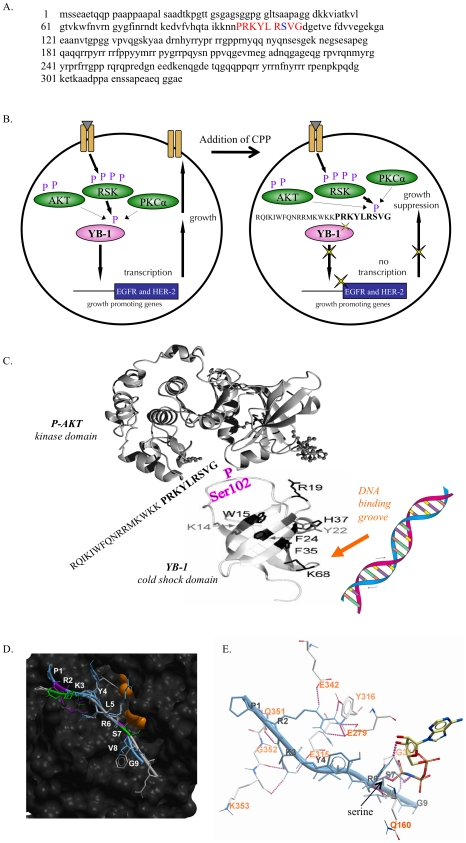
Schematic of the YB-1 peptide inhibitor mode-of-action. **A.** We designed a molecular decoy peptide of 9 amino acids (shown in red) that flanked the S102 site (shown in blue) on YB-1. The alignment of the peptide to the endogenous protein is shown. **B.** The YB-1 cell permeable peptide was designed to function in the following way. In cancer cells receptor tyrosine kinases initiate signaling events that culminate in the phosphorylation of YB-1^S102^ by RSK, PKCα and/or AKT. Once YB-1 is activated, it promotes the expression of growth receptors such as EGFR or HER-2. The CPP was therefore designed to enter cancer cells and serve as a sink for signaling via RSK, PKCα and AKT, since all of these kinases are potentially capable of phosphorylating YB-1. This CPP mimics the S102 site and thereby accepts the phosphate from adjacent ATP-binding site on the kinase, preventing endogenous YB-1 from becoming activated. In turn, growth-promoting genes are not transcribed, resulting in growth suppression. **C.** We predict that the S102 site of YB-1 lies in a flexible loop region of the cold-shock domain of YB-1. This region would be able to bind in a lock and key configuration to the peptide-binding site of the kinase domain of RSK, PKCα or AKT (shown). We hypothesize that this interaction promotes a conformational change in YB-1 that allows it to bind DNA and act as a transcription factor. The CPP would therefore, in theory, inhibit endogenous binding of YB-1. **D.** GSK3β, a known substrate of RSK, was used to further model how the CPP could align into the peptide-binding pocket of RSK, the predominate kinase that phosphorylates YB-1. GSK3β is shown in light grey with the CPP in blue overlaid on top in a peptide-binding pocket of RSK. The alignment was made manually so that the arginine (R) and serine (S) overlapped and would be in exact same conformation. The R and S of GSK3β are shown in green while the R and S of CPP are purple. ATP is shown in orange to demonstrate the relative positioning of the ATP-binding site. **E.** The CPP theoretically forms 16 H-bonds, shown as purple dashed lines, to the peptide-binding pocket of RSK. The serine site (arrow) is in a position to allow for a hydrogen bond with RSK as well as receive a phosphate group from ATP.

The transcription/translation factor YB-1 controls the expression of many growth regulatory and chemotherapy resistance genes and is thus an attractive target for cancer therapies. We designed a novel cell permeable peptide to competitively block phosphorylation of YB-1 at S102 (Figure 1). This 9-mer (Figure 1A) was tagged with an antennapedia leader sequence to facilitate cellular uptake and was initially biotin labeled for detection. Our objective was to produce an interference strategy whereby the CPP would serve as a molecular decoy to accept phosphorylation by kinases known to activate YB-1 at S102 such as RSK, PKCα and AKT [21]. Our working model was that the CPP would prevent phosphorylation of YB-1 and thereby block eventual transcriptional activation of key growth promoting genes such as EGFR or HER-2 (Figure 1B). This interference strategy should culminate in cancer cell growth suppression. Based on the crystal structure of the YB-1 cold-shock domain, we predicted that the S102 site would be located in a flexible loop region (Figure 1C) and that this area would fit into the peptide domains of RSK, PKCα or AKT in a lock and key configuration that would be disrupted via the interference peptide. Subsequently, YB-1 would be unable to bind to DNA (Figure 1C).

To build upon this concept we used computational modeling to show that the YB-1 peptide tethers to RSK. We chose to use RSK for the model because we previously published that YB-1 was efficiently phosphorylated by RSK1 and RSK2 [21]. A homology model of RSK was used to show how the peptide binds in a peptide-bound conformation and we used molecular docking to theoretically define the binding of the CPP to RSK. The peptide-binding site was adjacent to the ATP-binding pocket, with the CPP serine residue in a position to accept a phosphate group (Figure 1D). A space-filling model confirmed the CPP three-dimensional fit into the peptide-binding pocket of RSK, adjacent to this ATP-binding site (not shown). This RSK-YB-1 interaction was stabilized by 16 hydrogen bonds throughout the length of the peptide (Figure 1E, purple dotted lines; Table 1). For example, the R2 side chain of the CPP formed 5 hydrogen bonds with RSK (E342, E279 (3), Y316) and these were conserved in the solved AKT-GSK3β complex (reference complex not shown). Further, the peptide (catalytic) serine was in a position where it could form a hydrogen bond to the protein as well as receive a phosphate group from ATP (Figure 1E, arrow, indicated as S7). The ICM docking score of the RSK-YB-1 complex was -55, which indicated a high likelihood of a true binding interaction and was comparable to the score calculated for known AKT-GSK3β binding. This model therefore supports our previous work demonstrating that RSK phosphorylates YB-1 at S102 [21] and confirmed that the CPP could mimic this critical site. Thus, the competitive peptide should prevent the flexible loop region of endogenous YB-1 from binding to RSK.

**Table 1:** Theoretical H-bonds formed by the interaction between RSK and the CPP.

YB-1 CPP	RSK
P1	Q351
R2	E342, E279 (3), Y316
K3	Q351, K353, G352, E315
R6	Q160, AMP-PNP
S7	AMP-PNP (2)
V8	G312 (2)

**Figure 2:**
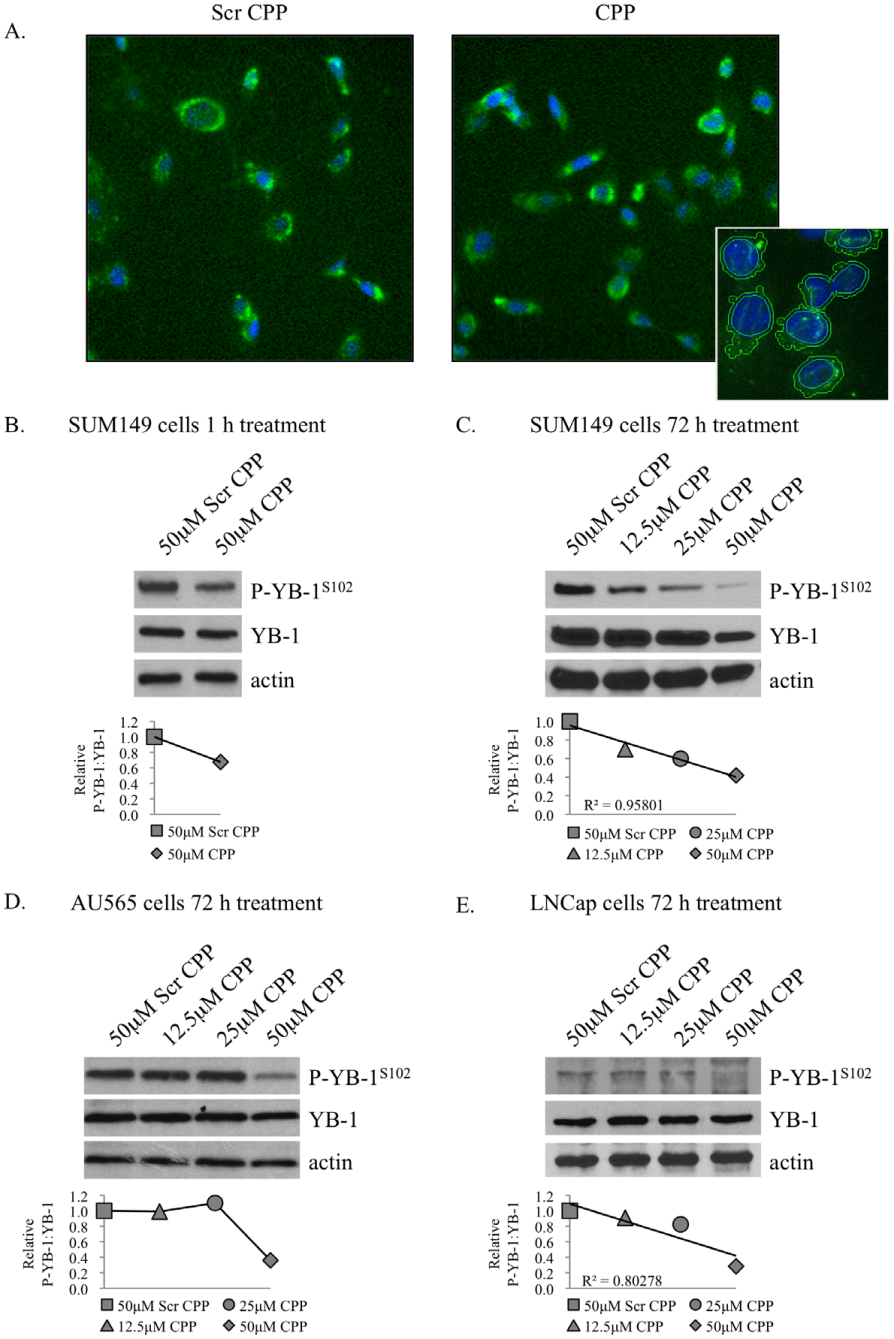
The CPP decreased YB-1 phosphorylation. **A.** To demonstrate the CPP entered cells, SUM149 cells were treated with either biotin-labeled scrambled CPP (1.25 µM, left panel) or CPP (1.25 µM, right panel) for 1 h and then visualized by detecting the biotin tag (green color). The nucleus was stained with Hoechst and is shown in blue. **B.** The CPP decreased P-YB-1^S102^ by 32% in as little as 1 h in SUM149 cells. **C–E.** Further, the CPP decreased phosphorylation of YB-1^S102^ following 72 h treatment in SUM149, AU565, and LNCap cells, respectively. Blots were quantified using ImageJ software and relative P-YB-1:YB-1 is shown below the western blots.

The CPP and scrambled control peptides were made cell permeable by fusing them to an antennapedia homeodomain sequence demonstrated to cross biological membranes [25]–[27]. Initially, they were biotin labeled to show that the scrambled control CPP and target CPP peptides rapidly entered SUM149 breast cancer cells to a similar degree within 1 hour where Oregon Green immunofluorescence was used to detect the peptides (Figure 2A). Once we determined that the CPP entered the cell, we next evaluated changes in signal transduction and determined that activation of YB-1^S102^ was blocked in as little as one hour in SUM149 cells (Figure 2B). Incubating cancer cells with increasing concentrations of the CPP for 72 h depressed P-YB-1^S102^ in SUM149, AU565 and LNCap cells, respectively (Figure 2C-E).

**Figure 3:**
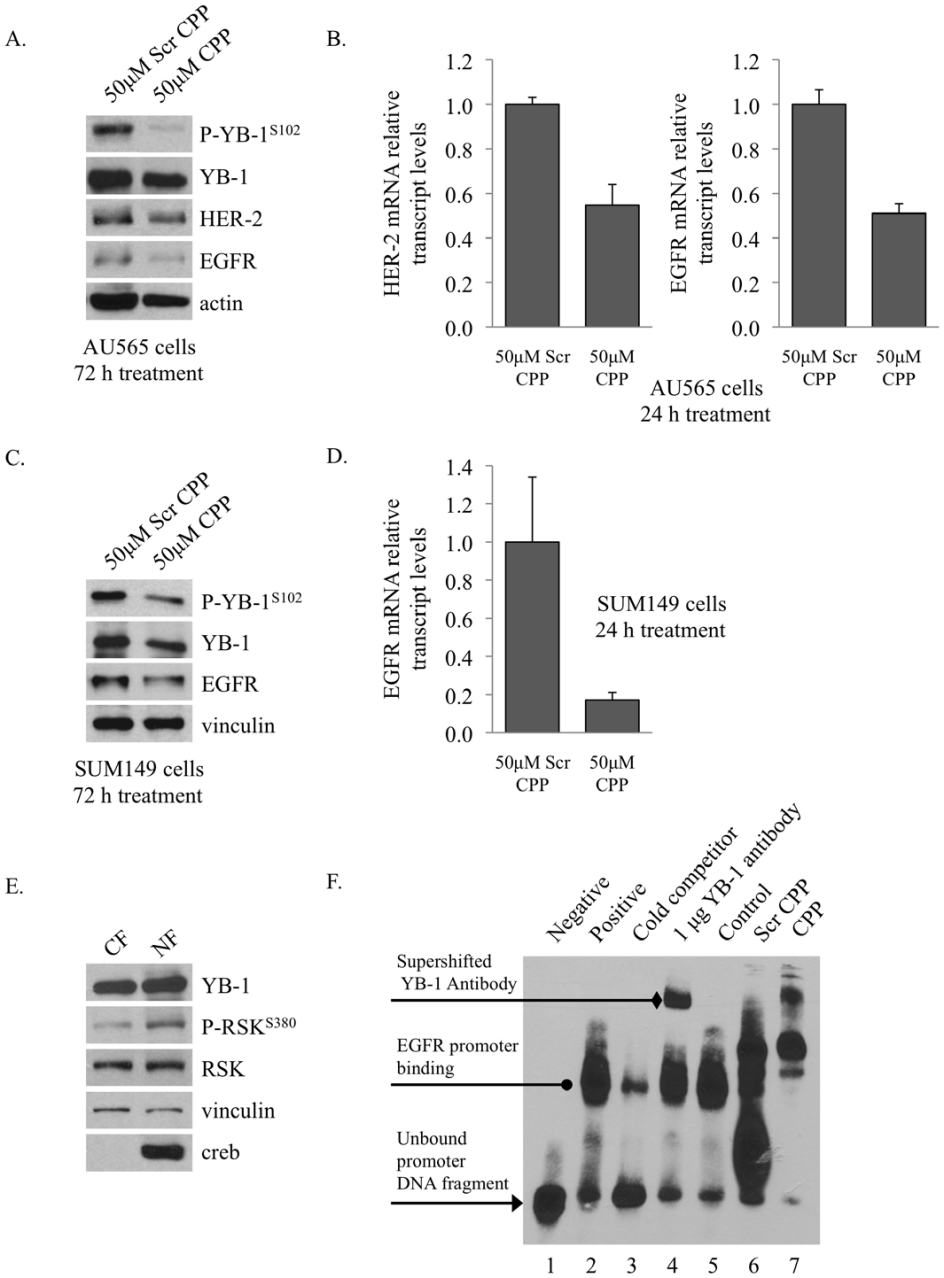
The CPP impaired YB-1s transcriptional activity against HER-2 and EGFR. **A.** Treating AU565 cells with the CPP for 72 h reduced P-YB-1^S102^ and that correlated with down-regulation of its target gene products EGFR and HER-2. **B.** HER-2 and EGFR transcripts are also markedly decreased in AU565 cells after 24 h CPP treatment based on qRT-PCR. * P<0.01. **C.** Likewise, the CPP decreased P-YB-1^S102^ and thus EGFR in SUM149 cells after 72 h. **D.** EGFR transcript levels were also decreased in the SUM149 cells following 24 h peptide treatment. * P<0.01. **E.** Nuclear extracts were prepared from SUM149 cells. A western blot confirmed that the nuclear fraction (NF) contained the components (YB-1 and RSK) necessary for the gel shift analysis. CF =  cytoplasmic fraction. **F.** Nuclear proteins from breast cancer cells, SUM149, bind to the EGFR promoter DNA (lane 2) compared to the unbound biotin labeled oligonucleotide DNA (lane 1). Binding was specific because cold competitive oligonucleotides to the same sequence diminished binding (lane 3). The transcription factor responsible for binding was YB-1 because introducing 1 µg of YB-1 antibody (lanes 4) caused a super-shift in the binding product. YB-1 bound with high affinity in the presence of acetic acid control (lane 5) and 25 µM scrambled peptide (lane 6), whereas incubation with 25 µM of the CPP blocked binding and cause a super-shift (lane 7).

Given this result, we next addressed whether blocking YB-1 activation with the CPP would impact the expression of its target genes HER-2 or EGFR. After 72 h, the CPP reduced HER-2 and EGFR protein levels in AU565 cells (Figure 3A). To further support this observation, we used quantitative real-time PCR to examine HER-2 and EGFR transcript levels and found that 24 h treatment with the CPP resulted in a ∼50% reduction of both (Figure 3B). Further, in SUM149 cells, EGFR protein and mRNA levels were decreased (Figure 3C-D). HER-2 was not detected because the SUM149 cells do not express this protein, likely due to differences in chromatin folding (Figure S1). We then extracted nuclear proteins from SUM149 cells to address whether the CPP interfered with YB-1's ability to bind to the EGFR promoter using gel shift assays. Prior to performing the gel shift, we determined that RSK was indeed present in the nuclear fraction and furthermore that it was activated based on S380 phosphorylation (Figure 3E). As expected, YB-1 was also abundantly found in the nuclear fraction (Figure 3E). The nuclear extracts containing endogenous RSK and YB-1 were incubated with 25 µM CPP and a gel shift assay was performed against the EGFR promoter in a region that we previously reported contains a key inverted CAAT region [10]. Nuclear proteins from SUM149 breast cancer cells bind to the EGFR promoter DNA compared to the unbound biotin labeled oligonucleotide DNA (Figure 3F, lane 1 vs. 2). Diminished binding was seen with cold competitive oligonucleotides, demonstrating that binding was specific (Figure 3F, lane 3). The addition of a YB-1 (1 µg) antibody caused a super-shift in the binding product, confirming that binding was due to this transcription factor (Figure 3F, lane 4). To show that the CPP could also cause a shift, nuclear extracts from SUM149 cells were pre-incubated with the solvent control, scrambled peptide, or the CPP. YB-1 bound with high affinity in the presence of diluent or scrambled peptide (Figure 3F, lanes 5 and 6) whereas incubation with 25 µM of the CPP blocked binding (Figure 3F, lane 7). These results were independently confirmed with 25 µM and 50 µM of the CPP in a gel shift using SUM149 nuclear extracts (data not shown). Taken together, these data show that the CPP successfully blocked the activation of YB-1 and thereby prevented its binding to the EGFR promoter. Further, this result supports our previous work showing that YB-1 binds to EGFR in a S102 dependent manner [10] and that the MAPK inhibitor PD098059 or the RSK inhibitor SL0101 reverse YB-1 binding to the EGFR promoter [21].

**Figure 4:**
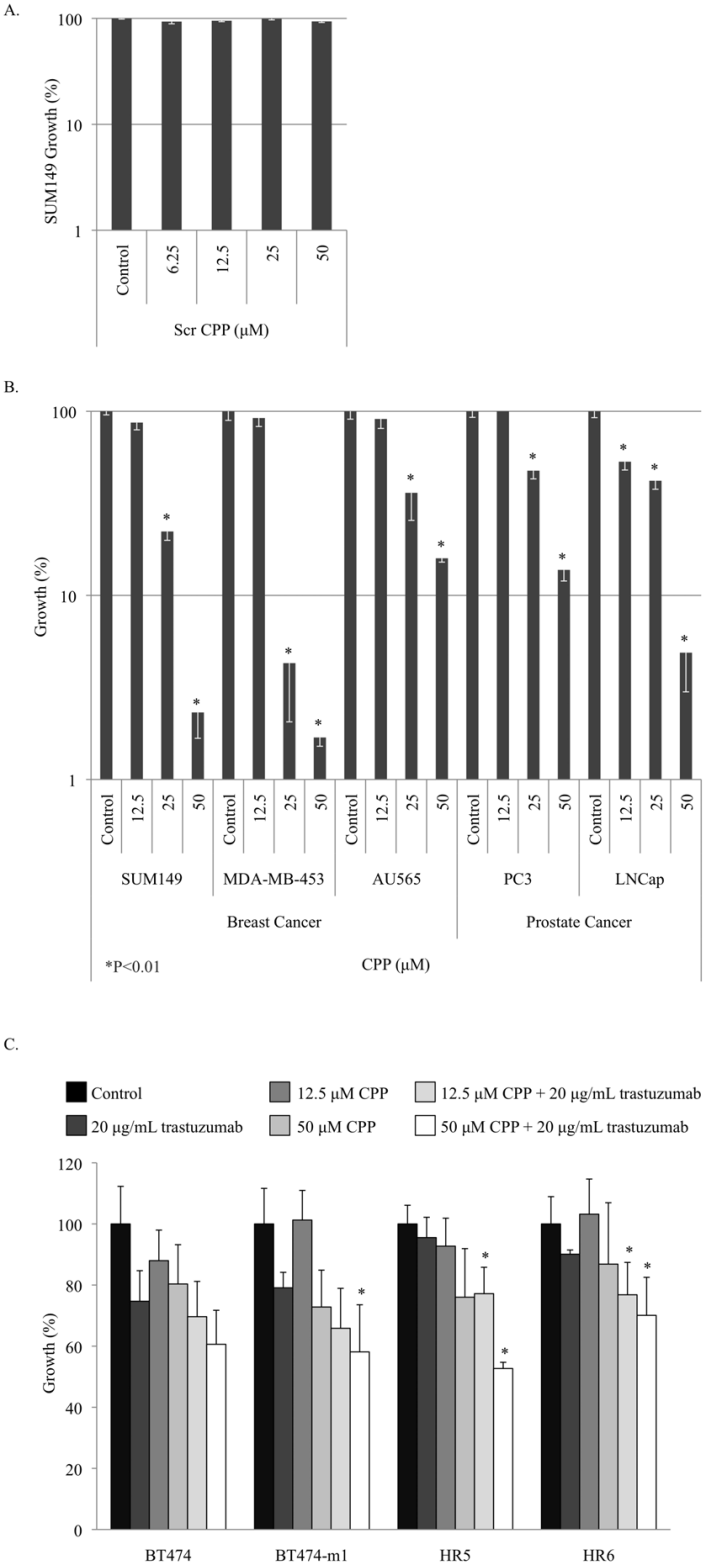
The cell permeable peptide inhibited growth of breast and prostate cancer cell lines. **A.** The scrambled CPP had no effect on the growth of breast cancer SUM149 cells following exposure for 72 h. **B.** The CPP inhibited the growth of breast (SUM149, MDA-MB-453 and AU565) and prostate cancer cells (PC3 and LNCap) cell lines in a dose dependent manner after 72 h. *P<0.01 compared to control lane. Each of the cell lines was tested with the peptides in replicates of three on two separate occasions. **C.** The CPP also suppressed the growth of BT474 and its metastatic variant the BT474-m1 cells. Notably it increased the sensitivity of these cells to trastuzumab (20 µg/ml) following 72 h treatment. Further, treating the trastuzumab resistant variants HR5 and HR6 with the CPP allowed the cells to overcome drug insensitivity. In all cell lines, the combination of CPP and trastuzumab significantly (P<0.01) decreased growth compared to the control lane. Indicated (*) combinations were also significant (P<0.01) compared to trastuzumab treatment alone.

We next questioned whether the CPP would preferentially inhibit tumour cell growth while sparing normal cells. The scrambled CPP had no effect on growth (Figure 4A) while, consistent with an attenuation in signaling, the CPP inhibited growth in a dose-dependent manner of breast (SUM149, MDA-MB-453, AU565) and prostate (PC3, LNCap) cancer cell lines (Figure 4B). In most cases, growth inhibition was >90% at the highest dose of 50 µM. We next determined that the addition of the CPP sensitized the HER-2 over-expressing cells BT474 and BT474-m1's (a metastatic variant of the former) to trastuzumab (Herceptin®) (Figure 4C). The cells were treated for 72 h with trastuzumab (20 µg/ml), the CPP (12.5 or 50 µM) or a combination of the two. Additionally, we challenged the trastuzumab resistant variants HR5 and HR6 [34] cells with the CPP and found that it was able to significantly suppress growth in these otherwise recalcitrant cell lines (Figure 4C).

**Figure 5:**
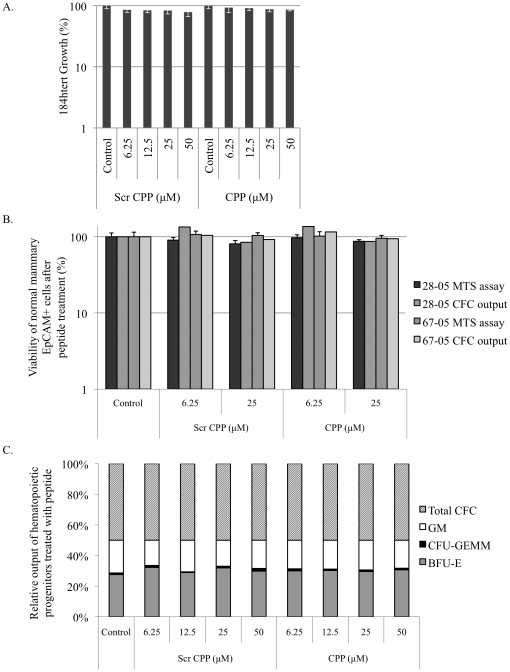
The CPP had no growth effect on normal breast epithelial cells or hematopoietic progenitors. **A.** Neither the scrambled CPP nor the CPP affected the growth of normal immortalized human epithelial 184htert cells after being exposed to the peptide for 72 h based on MTS proliferation assays. **B.** Normal breast epithelial cells were taken from two women (coded as 28-05 and 67-05) who had undergone a reduction mammoplasty. The cells were isolated and treated with either the scrambled peptide or CPP (6 or 25 µM) for 72 h and then assessed for viability using an MTS assay. These patients were also evaluated using the same amount of the peptides in a 7-day CFC output assay. **C.** Relative output of hematopoietic progenitors remained unchanged with CPP treatment. CFC assays of CD34+ bone marrow cells were performed with various concentrations of scrambled CPP or CPP. Erythroid colonies (BFU-Es), granulopoietic colonies (CFU-GMs) and multi-lineage colonies (CFU-GEMM) were scored by direct visualization using an inverted microscope (32×) after 14 days using standard criteria. These values were then added together to obtain total CFC counts per cells plated and expressed as percent of control.

By contrast, neither the scrambled control peptide nor the CPP inhibited growth of normal human mammary epithelial cells (184htert) (Figure 5A). The lack of activity in the 184htert cells suggest that the peptide does not have unwarranted off-target effects given the observation that these cells do not express YB-1 [30] (Figure S1). This result suggested that the growth inhibitory effect of the CPP in cancer cells depends upon the expression of YB-1. It has previously been shown that YB-1 is undetectable in normal breast tissue [39] or in normal bone marrow plasma cells [40]. To further determine the potential safety of this peptide, we report that neither the scrambled peptide nor the CPP had any growth effect on normal breast epithelial cells taken from reduction mammoplasties based on MTS as well as CFC assays (Figure 5B). Secondly, in a hematopoietic progenitor assay, differentiation of normal blood progenitor cells was not altered by the CPP (Figure 5C). Taken together, these assays illustrate that the CPP caused a growth inhibition in cancer cells but not in normal cells.

Cancer cells are generally thought to have hijacked normal control mechanisms that regulate proliferation and survival. RSK itself has been shown to be an important regulator of proliferation [23], [24] and our data supports the hypothesis that blocking RSK with the CPP causes growth suppression. In recent years, directly targeting cell-signaling pathways to inhibit cancer growth has gained momentum, with the development of rationally designed peptide inhibitors, monoclonal antibodies and small molecules. Interference peptides that target oncogenic pathways, such as the CPP described herein, may have broad utility in cancer therapeutics, or, at the very least, substantiate promising therapeutic targets. Examples such as shepherdin, a peptidomimetic that blocks the interaction of survivin and Hsp90, causing massive apoptosis in cancer cells, highlights the feasibility of such an approach [27], [41]. This anti-cancer agent is remarkably selective with high affinity to cancer cells, well tolerated *in vivo*, and non-toxic to normal cells. Shepherdin is also more potent than other Hsp90 inhibitors, validating this more targeted approach. The on-going development of other cell penetrating peptide inhibitors against factors such as NF-κB [42] and c-Myc [43], among others, demonstrates that these directed therapies comprise an active area of research [44], [45]. Further, efforts to stabilize peptides through chemical modifications such as peptide stapling promises to expand the repertoire of achievable therapies [46]. Our work with the CPP suggests that the YB-1 axis is also a promising target for cancer therapeutics and we are actively pursuing this lead.

## Supporting Information

Figure S1 Cell line western blots. A. Normal immortalized mammary epithelial cells (184htert cells) do not express YB-1 although they do have RSK and AKT. A and B. The cancer cells lines used herein express YB-1, P-YB-1 and the activating kinases RSK and AKT.
(0.65 MB TIF)
