# Supplementary figures and images for: Molecular Decoy to the Y-Box Binding Protein-1 Suppresses the Growth of Breast and Prostate Cancer Cells whilst Sparing Normal Cell Viability
Source: PLoS One. 2010 Sep 10;5(9):e12661. doi: 10.1371/journal.pone.0012661 (PMC2937023; doi:10.1371/journal.pone.0012661)

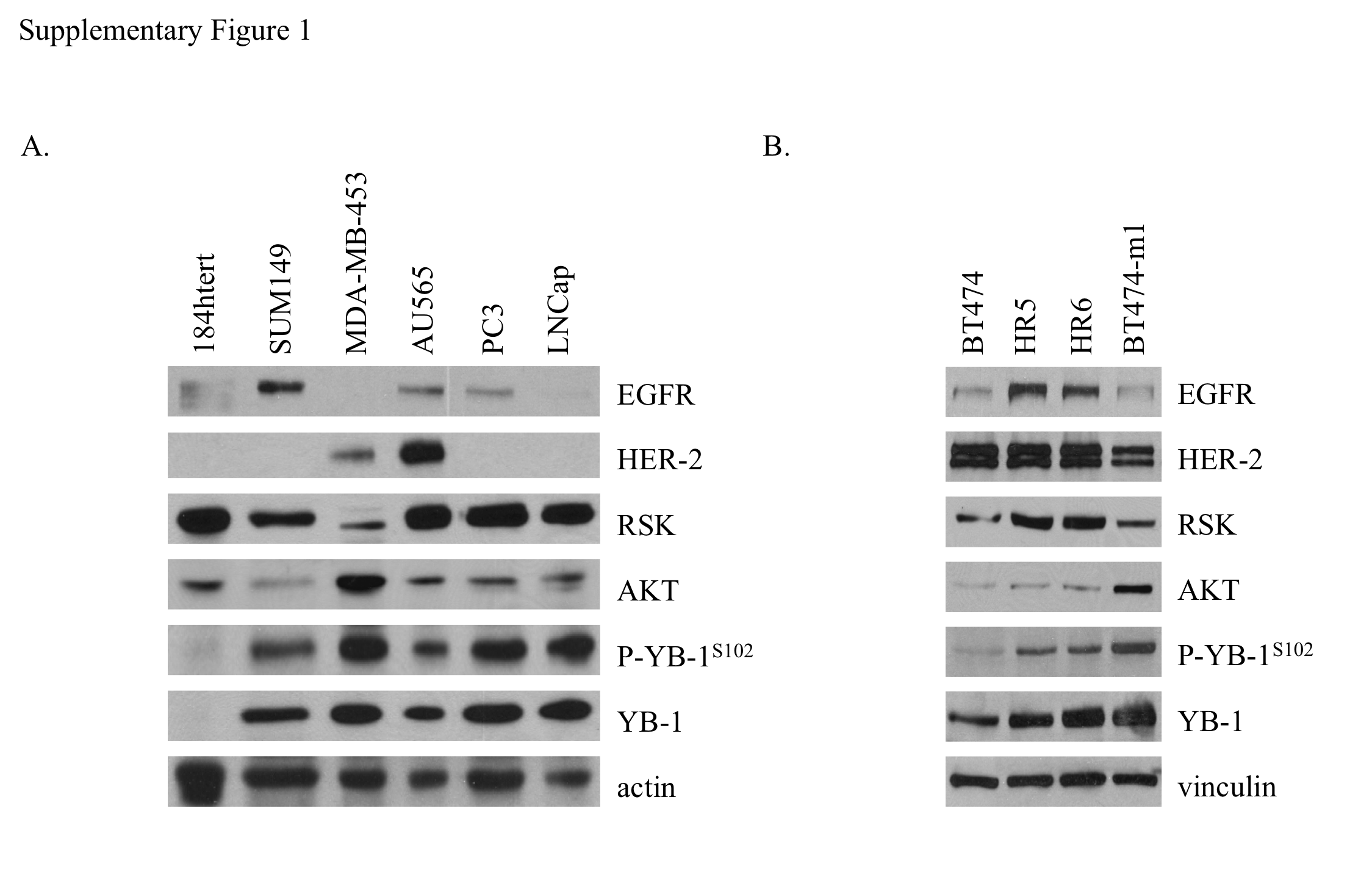

Supplement: Figure S1 — Cell line western blots. A. Normal immortalized mammary epithelial cells (184htert cells) do not express YB-1 although they do have RSK and AKT. A and B. The cancer cells lines used herein express YB-1, P-YB-1 and the activating kinases RSK and AKT. (0.65 MB TIF) [file pone.0012661.s001.tif]
